# Gonorrhœal Peritonitis

**Published:** 1888-11

**Authors:** 


Gonorrhœal Peritonitis.—Dr. E. Ceppi, of Porrentruy (*Revue Médicale de la Suisse Romande*), reports a case of chronic purulent peritonitis in a previously healthy and robust woman, aged twenty-nine, which was cured by abdominal incision and drainage. The disease proved to be of gonorrhœal origin, small accumulations of Neisser's gonococci being found in the pus-cells. Similar microbes were discovered also in the purulent discharge from the uterus. This case is said to be the first in which the gonococcus has been found in the peritonitic exudation.—*N. Y. Med. Record.*

